# Clinical applications and limitations of large language models in nephrology: a systematic review

**DOI:** 10.1093/ckj/sfaf243

**Published:** 2025-09-18

**Authors:** Zoe Unger, Shelly Soffer, Orly Efros, Lili Chan, Eyal Klang, Girish N Nadkarni

**Affiliations:** First Faculty of Medicine, Charles University, Prague, Czech Republic; Institute of Hematology, Davidoff Cancer Center, Rabin Medical Center, Petah-Tikva, Israel; School of Medicine, Tel Aviv University, Tel Aviv, Israel; School of Medicine, Tel Aviv University, Tel Aviv, Israel; National Hemophilia Center and Thrombosis Institute, Sheba Medical Center, Ramat Gan, Israel; Division of Data-Driven and Digital Medicine (D3M), Icahn School of Medicine at Mount Sinai, New York, NY, USA; Charles Bronfman Institute of Personalized Medicine, Icahn School of Medicine at Mount Sinai, New York, NY, USA; Barbara T. Murphy Division of Nephrology, Icahn School of Medicine at Mount Sinai, New York, NY, USA; Division of Data-Driven and Digital Medicine (D3M), Icahn School of Medicine at Mount Sinai, New York, NY, USA; Charles Bronfman Institute of Personalized Medicine, Icahn School of Medicine at Mount Sinai, New York, NY, USA; Division of Data-Driven and Digital Medicine (D3M), Icahn School of Medicine at Mount Sinai, New York, NY, USA; Charles Bronfman Institute of Personalized Medicine, Icahn School of Medicine at Mount Sinai, New York, NY, USA; Barbara T. Murphy Division of Nephrology, Icahn School of Medicine at Mount Sinai, New York, NY, USA

**Keywords:** artificial intelligence, kidney disease, large language models, nephrology

## Abstract

**Background:**

Large language models (LLMs) have emerged as potential tools in healthcare. This systematic review evaluates the applications of text-generative conversational LLMs in nephrology, with particular attention to their reported advantages and limitations.

**Methods:**

A systematic search was performed in PubMed, Web of Science, Embase and the Cochrane Library in accordance with the Preferred Reporting Items for Systematic Reviews and Meta-Analyses guidelines. Eligible studies assessed LLM applications in nephrology. PROSPERO registration number CRD42024550169.

**Results:**

Of 1070 records screened, 23 studies met inclusion criteria, addressing four clinical applications in nephrology. In patient education (*n* = 13), GPT-4 improved the readability of kidney donation information from a 10th to a 4th grade level (9.6 ± 1.9 to 4.30 ± 1.71) and Gemini provided the most accurate answers to chronic kidney disease questions (Global Quality Score 3.46 ± 0.55). Regarding workflow optimization (*n* = 7), GPT-4 achieved high accuracy (90–94%) in managing continuous renal replacement therapy alarms and improved diagnosis of diabetes insipidus using chain-of-thought and retrieval-augmented prompting. In renal dietary guidance (*n* = 2), Bard AI led in classifying phosphorus and oxalate content of foods (100% and 84%), while GPT-4 and Bing Chat were most accurate for potassium classification (81%). For laboratory data interpretation (*n* = 1), Copilot significantly outperformed ChatGPT and Gemini in simulated nephrology datasets (median scores 5/5 compared with 4/5 and 4/5; *P* < .01). TRIPOD-LLM assessment revealed frequent omissions in data handling, prompting strategies and transparency.

**Conclusions:**

While LLMs may enhance various aspects of nephrology practice, their widespread adoption remains premature. Input-quality dependence and limited external validation restrict generalizability. Further research is needed to confirm their real-world feasibility and ensure safe clinical integration.

KEY LEARNING POINTS **What was known:**

- Large language models (LLMs) such as ChatGPT may process medical data to support clinical workflows and answer medical questions.
- Artificial intelligence–based tools have shown promise in various medical fields, but their specific value in nephrology remains under investigation.

**This study adds:**

- We provide a consolidated overview of current LLM applications across four key domains of nephrology.
- It is essential that LLMs undergo rigorous external validation prior to widespread clinical adoption.

**Potential impact:**

- LLMs may streamline clinical processes and support guideline-concordant diagnosis.
- They could also improve patient education and promote healthcare equity.

## INTRODUCTION

Large language models (LLMs) such as ChatGPT [1] are advanced artificial intelligence (AI) systems that generate human-like text [2]. Emerging evidence suggests that these models have potential in various medical specialties [3–11]. The complex nature of kidney diseases and their treatment could make LLM technology useful for improving clinical management.

LLMs generate complex language by predicting the most probable word sequences. They represent an advanced form of deep learning, an AI approach inspired by neuronal connections; at their core, each unit functions similar to a logistic regression node [1, 12–14]. Table 1 provides a concise overview of AI modalities and Supplementary Material 1 presents a more detailed discussion.

**Table 1:** Overview of AI modalities.

Modality	Definition
Artificial intelligence (AI)	A field that trains computers to perform tasks requiring human-like cognition
Natural language processing (NLP)	A branch of AI focused on enabling computers to understand, process and generate human language
Deep learning (DL)	A subset of AI using a multilayered architecture to process complex data, including language and visual inputs
Transformer	System that converts, transfers or modifies inputs from one form to another. In AI, transformers are DL models that use attention mechanisms for contextual understanding
Bidirectional encoder representations from transformers (BERT)	A transformer-based model that enhances NLP tasks by analysing text bidirectionally for improved contextual comprehension
Large language models (LLMs)	Advanced AI models trained on large datasets, forming the foundation of modern chatbots and enabling refined, context-aware language generation

Multimodal LLMs may facilitate the interpretation of complex datasets, including medical images [15, 16]. They could also tailor treatment recommendations by aggregating evidence-based literature [17]. Furthermore, LLMs may assist with routine tasks such as documenting medical records, analysing laboratory results and interpreting different imaging studies [18–20]. Such automation has the potential to reduce administrative burden and streamline clinical processes, yet its direct effect on patient-centred outcomes in nephrology has not been confirmed.

**Figure 1:**
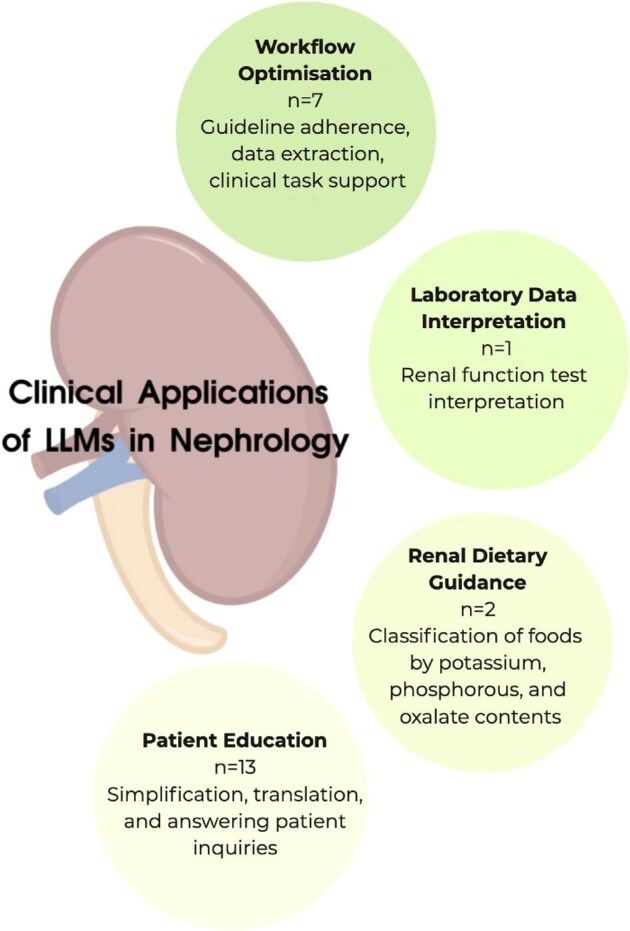
The current clinical applications of conversational LLMs in nephrology.

In this review we summarize the current clinical applications of conversational LLMs in nephrology (Fig. 1). We also critically evaluate their capabilities and limitations in kidney disease management.

## MATERIALS AND METHODS

### Search strategy

**Figure 2:**
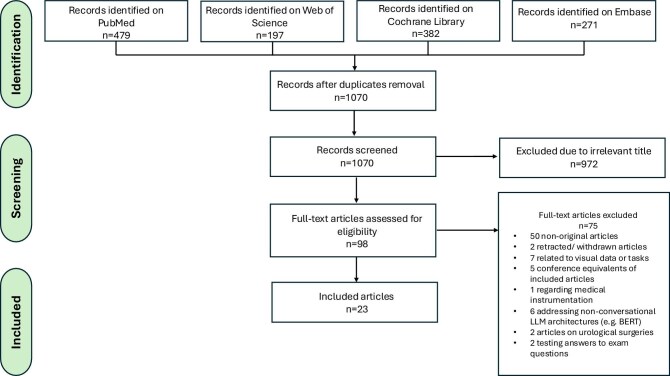
Search and selection flow chart.

This systematic review was conducted in accordance with the Preferred Reporting Items for Systematic Reviews and Meta-Analyses (PRISMA) guidelines (Fig. 2). A literature search was conducted in MEDLINE (PubMed), Web of Science, Embase and the Cochrane Library on 21 July 2024. Search terms covered two domains—nephrology and LLMs. Table 2 summarizes the search terms and the complete search methodology is described in Supplementary Material 2.

**Table 2:** Search terms.

Search block (joined by AND)	Keywords (joined by OR)
Nephrology	Kidney, renal, nephrology, kidney disease, renal disease, renal disorder, nephropathy, acute kidney injury, acute renal failure, AKI, acute renal insufficiency, chronic kidney disease, CKD, chronic renal failure, chronic renal insufficiency, haemodialysis, dialysis, renal replacement therapy, glomerulonephritis, glomerulopathy, glomerular disease, glomerular disorder, nephrotic syndrome, nephritic syndrome, urinalysis, renal function test
LLMs	Large language model, LLM, ChatGPT, OpenAI, Microsoft Bing, Google Bard, Google Gemini, BERT, transformers

Studies were included if they investigated clinical applications of LLMs in nephrology or kidney diseases. To meet the inclusion criteria, studies had to be peer-reviewed original research articles published in English and directly relevant to LLM integration in nephrology practice, including aspects such as patient care, diagnostic processes, treatment efficacy or clinical outcomes.

Exclusion criteria were applied to maintain relevance and methodological quality. Non-original articles, including reviews, editorials and commentaries, were excluded. Studies that focused on areas outside of nephrology—primarily urologic surgery, clinical pharmacology and laboratory instrumentation—were omitted. Research centred on LLM optimization, engineering applications or other technological advances without an explicit link to patient care or clinical outcomes in nephrology was also excluded. Articles evaluating AI tools in non-clinical settings (professional certification assessments, literature search assistance, scientific writing support or exam question responses) did not meet the inclusion criteria. Finally, we excluded studies that focused on non-LLM AI, transformer-based models without conversational capabilities [e.g. bidirectional encoder representations from transformers (BERT)] or the use of LLMs for visual-data processing.

This systematic review is registered in PROSPERO (CRD42024550169).

### Study selection

Two reviewers (Z.U. and S.S.) independently screened the titles and abstracts against the inclusion criteria. A further review of the full-text articles was performed when eligibility was uncertain. A third reviewer (E.K.) resolved any disagreements in the study selection process.

### Data extraction

Data were collected using a standardized data extraction form. The following variables were recorded: year of publication, study design, study location, ethical approval, sample size, inclusion and exclusion criteria, population description, use and size of any online database, presence of an independent test dataset, clinical application, evaluation metrics and performance outcomes.

### Quality assessment, risk of bias and reporting standards evaluation

Risk of bias was assessed with an adapted version of the Quality Assessment of Diagnostic Accuracy Studies 2 (QUADAS-2) tool [21]. Compliance with reporting standards was evaluated with the Transparent Reporting of a Multivariable Model for Individual Prognosis Or Diagnosis–Large Language Models (TRIPOD–LLM) checklist [22].

## RESULTS

### Study selection and classification process

Our literature search identified 1070 records. Of these, 23 studies met the inclusion criteria (Fig. 2). We grouped the eligible studies into four clinical application domains in nephrology: workflow optimization, laboratory data interpretation, renal dietary guidance and patient education (Table 3).

**Table 3:** Categorization of included articles.

Clinical application	Title	Author	Journal	Year of publication
Workflow optimization	Personalized medicine transformed: ChatGPT's contribution to continuous renal replacement therapy alarm management in intensive care units	Sheikh *et al.* [23]	*J Pers Med*	2024
	How to improve ChatGPT performance for nephrologists: a technique guide	Miao *et al.* [24]	*J Nephrol*	2024
	ChatGPT and artificial intelligence in transplantation research: is it always correct?	Rawashdeh *et al.* [25]	*Cureus*	2023
	Enhanced artificial intelligence strategies in renal oncology: iterative optimization and comparative analysis of GPT 3.5 versus 4.0	Liang *et al.* [26]	*Ann Surg Oncol*	2024
	Evaluation of ChatGPT for patient counseling in kidney stone clinic: a prospective study	Javid *et al.* [27]	*J Endourol*	2024
	Comparative analysis of artificial intelligence chatbot recommendations for urolithiasis management: a study of EAU guideline compliance	Altintas *et al.* [28]	*Fr J Urol*	2024
	The efficacy of artificial intelligence in urology: a detailed analysis of kidney stone-related queries	Cil *et al.* [29]	*World J Urol*	2024
Laboratory data interpretation	Response accuracy of ChatGPT 3.5 Copilot and Gemini in interpreting biochemical laboratory data a pilot study	Kaftan *et al.* [19]	*Sci Rep*	2024
Renal dietary guidance	AI-powered renal diet support: performance of ChatGPT, Bard AI, and Bing Chat	Qarajeh *et al.* [32]	*Clin Pract*	2023
	Personalized medicine in urolithiasis: AI chatbot-assisted dietary management of oxalate for kidney stone prevention	Aiumtrakul *et al.* [33]	*J Pers Med*	2024
Patient education	Empowering inclusivity: improving readability of living kidney donation information with ChatGPT	Garcia Valencia *et al.* [34]	*Front Digit Health*	2024
	AI-driven translations for kidney transplant equity in Hispanic populations	Garcia Valencia *et al.* [35]	*Sci Rep*	2024
	Using ChatGPT for kidney transplantation: perceived information quality by race and education levels	Lee *et al.* [36]	*Clin Transplant*	2024
	Can large language models provide accurate and quality information to parents regarding chronic kidney diseases?	Naz *et al.* [37]	*J Eval Clin Pract*	2024
	Evaluating ChatGPT's accuracy in responding to patient education questions on acute kidney injury and continuous renal replacement therapy	Sheikh *et al.* [38]	*Blood Purif*	2024
	Availability of ChatGPT to provide medical information for patients with kidney cancer	Choi *et al.* [39]	*Sci Rep*	2024
	ChatGPT: is this patient education tool for urological malignancies readable for the general population?	Thia *et al.* [40]	*Res Rep Urol*	2024
	How well do artificial intelligence chatbots respond to the top search queries about urological malignancies?	Musheyev *et al.* [41]	*Eur Urol*	2024
	Quality of information about kidney stones from artificial intelligence chatbots	Musheyev *et al.* [42]	*J Endourol*	2024
	Urological cancers and ChatGPT: assessing the quality of information and possible risks for patients	Ozgor *et al.* [43]	*Clin Genitourin Cancer*	2024
	Application of artificial intelligence to patient-targeted health information on kidney stone disease	Kianian *et al.* [44]	*J Ren Nutr*	2024
	Accuracy and readability of kidney stone patient information materials generated by a large language model compared to official urologic organizations	Halawani *et al.* [45]	*Urology*	2024
	Evaluating the performance of ChatGPT in answering questions related to urolithiasis	Cakir *et al.* [46]	*Int Urol Nephrol*	2024

The characteristics of the included studies are summarized in Table 4. A task-specific performance comparison is presented in Table 5, and Table 6 synthesizes the reported advantages and limitations of each tested LLM modality.

**Table 4:** Characteristics of included articles.

Clinical application	Ref.	Model	Objective	Training set	Reference standard	Sample size	Key findings
Workflow optimization	[23]	ChatGPT, GPT-4	Compare accuracy in CRRT alarm management	N/A	Nephrologist answer key	50 CRRT alarm questions	GPT-4 achieved 90–94% accuracy, outperforming ChatGPT (84–86%)
	[24]	Custom GPT-4	Diagnose nephrogenic diabetes insipidus using CoT and RAG prompting	N/A	Standard prompting	Unspecified	CoT provided more specificity; RAG prompting aligned GPT-4 responses with KDIGO guidelines
	[25]	ChatGPT	Answer kidney transplant treatment-related questions	N/A	Specialists	4 questions	Responses requiring deeper knowledge contained inaccuracies; answers were nearly identical across sessions
	[26]	ChatGPT, GPT-4	Answer binary clinical questions about RCC before and after iterative fine-tuning	Question variants, using command-line interface	Guidelines	80 RCC questions by urologists	GPT-4 had higher accuracy than ChatGPT (77.5% and 67.08%, respectively; *P* < .05). Fine-tuning the Turbo model increased ChatGPT's accuracy
	[27]	ChatGPT	Compare kidney stone patient counselling with that provided by practicing urologists	N/A	Experienced urologists	61 questions from 12 kidney stone patients	ChatGPT outperformed urologists (*P* < .001) in accuracy, empathy, completeness and practicality. IRR was low; only empathy showed significant agreement (κ = 0.163)
	[28]	GPT-4, Perplexity, Bing, Bard AI	Medical recommendations on urolithiasis in alignment with EAU 2023 guidelines	N/A	Urologist's rating in compliance with EAU guidelines	115 questions based on EAU urolithiasis guidelines	GPT-4 and perplexity outperformed Bing and Bard (*P* < .001) with mean scores of 4.80 ± 0.47 and 4.68 ± 0.80, respectively
	[29]	GPT-4	Evaluate the LLM’s performance in answering kidney stone questions and its adaptability over time	N/A	EAU 2023 guidelines	90 questions by endourologists, 50% binary and 50% elaborative	High accuracy: correct rates were 80% and 93.3% for binary and descriptive questions, respectively
Laboratory data interpretation	[19]	ChatGPT, Copilot, Gemini	Interpret renal function test results along with other lab data	N/A	Independent physician ratings	10 simulated laboratory datasets	Copilot achieved the highest accuracy (median score = 5), significantly outperforming other models (*P* < .01)
Renal dietary guidance	[32]	ChatGPT, GPT-4, Bard AI, Bing Chat	Classify food items based on potassium and phosphorus content	N/A	Mayo Clinic Renal Diet Handbook	240 food items selected	GPT-4 and Bing Chat had the highest potassium classification accuracy (81%); Bard AI was most accurate for phosphorus (100%)
	[33]	ChatGPT, GPT-4, Bard AI, Bing Chat	Categorize food items by oxalate content	N/A	Mayo Clinic Oxalate Diet Handbook	539 food items	Bard AI showed significantly higher accuracy of 84% (*P* < .001), regardless of oxalate levels, followed by Bing (60%), GPT-4 (52%), and ChatGPT (49%), showing decreasing accuracy with increased oxalate content
Patient education	[34]	ChatGPT, GPT-4	Simplify FAQs on kidney donation	N/A	Donate Life America website	27 FAQs	GPT-4 reduced readability to 4.30 ± 1.71, outperforming other models, with 96.3% success versus ChatGPT's 59.26%
	[35]	ChatGPT, GPT-4	Translate kidney transplant FAQs into Spanish	N/A	Spanish-speaking nephrologist ratings	54 FAQs on kidney transplant (sources: OPTN, NHS and NKF)	Both models had high accuracy (ChatGPT: 4.89 ± 0.31, GPT-4: 4.94 ± 0.23) and cultural sensitivity (4.96 ± 0.19), with no significant difference (*P* > .05)
	[36]	ChatGPT	Answered kidney transplant questions; responses were evaluated in an online survey	N/A	565 individuals	86 questions based on 4624 Reddit posts	Higher education levels predicted higher perceived quality among non-White participants (mean = 6.03, SE = 0.39) but lower perceived quality among White participants (mean = 5.85, SE = 0.39)
	[37]	ChatGPT, Gemini, Copilot	Answer CKD FAQs	N/A	Paediatric nephrologist ratings (based on KDIGO guidelines)	40 FAQs (online sources unspecified)	Gemini provided the most accurate CKD information (GQS 3.46 ± 0.55) compared with other models
	[38]	GPT-4	Answered AKI and CRRT questions in various linguistic formats	N/A	Critical care nephrologist evaluation	89 questions from Mayo Clinic Handbook (50 on CRRT, 39 on AKI)	98% accuracy for CRRT questions with misspellings or incomplete sentences, 97% for all formats on AKI
	[39]	ChatGPT	Evaluation of responses to common questions on kidney cancer	N/A	Survey responses of 24 urologists, 9 of which are kidney cancer experts	10 questions based on Google searches	Only 29.2% of raters believed that ChatGPT could replace a urologist's explanation. Overall positive evaluation of the responses was 77.9%
	[40]	ChatGPT	Assessment of readability of information regarding kidney cancer (among others)	N/A	Cancer Council Australia, UpToDate, Mayo Clinic	Unspecified number of questions, based on patient inquiries	No statistically significant difference in readability between ChatGPT and the reference standards (*P* > .05). When targeting 16-year-olds, ChatGPT responses showed a significant improvement of 16.25 ± 12.05 points
	[41]	ChatGPT, Perplexity, Chat Sonic, Bing AI	Assessment of information quality on kidney cancer (among others)	N/A	National Comprehensive Cancer Network guidelines	5 most common searches in Google Trends	Moderate to high quality of health information with median DISCERN of 4/5 and no misinformation with median 1/5. Relatively difficult readability (11th- to 12^th^-grade reading level)
	[42]	ChatGPT, Perplexity, Chat Sonic, Bing AI	Assessment of kidney stone information quality	N/A	2 researchers	5 most common searches in Google Trends, and 6 headers from NIDDK website	High-quality health information with median DISCERN of 4/5 and no misinformation with median 1/5. Relatively difficult readability (11^th^-grade reading level)
	[43]	GPT-4	Quality assessment of responses to kidney cancer questions	N/A	3 uro-oncologists	47 FAQs from various sources and 37 EAU guideline-based questions	Kidney cancer FAQs had GQS of 5/5 in 68.1% of cases. GQS scores for FAQs were significantly higher than for EAU guideline questions (4.5 ± 0.8 and 3.7 ± 1.0, respectively; *P* < .01)
	[44]	ChatGPT	Assess the readability and quality of LLM responses comparing to online search results	N/A	Webpages from Google, Bing and Yahoo (evaluated by 2 researchers)	Kidney stone prevention: 50 webpages and 1 LLM response; kidney stone treatment: 49 webpages and 1 LLM response	ChatGPT showed readability level within the AMA guidelines: scored as 5th-grade level, while the online search results were of 10th–12th grade
	[45]	ChatGPT, GPT-4	Assess the readability and accuracy of information on kidney stone provided by LLMs, comparing with official associations	N/A	AUA, CUA and EAU (evaluated by 2 researchers)	Top 10 FAQs on Google	Both ChatGPT and GPT-4 showed inferior readability compared with official associations (grade levels: ChatGPT 13–14, GPT-4 11–13, CUA 10–12, EAU 9–11, AUA 8–10)
	[46]	ChatGPT	Answer inquiries on kidney stones	N/A	2 urologists	93 FAQs from official websites and social media, and 60 questions based on EAU guideline	94.6% of FAQs and 83.3% of guideline-based questions were completely correct

GPT: generative pretrained transformer; N/A: not applicable; RCC: renal cell carcinoma; IRR: interrater reliability; EAU: European Association of Urology; OPTN: Organ Procurement and Transplantation Network; NHS: National Health Service; NKF: National Kidney Foundation; NIDDK: National Institute of Diabetes and Digestive and Kidney Diseases; AMA: American Medical Association; AUA: American Urological Association; CUA: Canadian Urological Association.

**Table 5:** Comparison of task-specific LLM performance.

Model	Accuracy	Readability	Information Quality	Cultural Sensitivity	Empathy/Communication	Guideline Alignment	Learning/Adaptability	Others
ChatGPT	CRRT alarms: 85% [23] RCC Qs: 67.08%, 93.75–100% (fine-tuned/iterative) [26] Kidney cancer Qs: 77.9% positive evaluation rate [39] Kidney stone Qs: 83.3% and 94.6% [46]; outperformed urologists by mean accuracy difference of 6.9–7.34 (*P* < .001) [27] Kidney stone information: 2–3/5 [45] Oxalate ranking: 49% [33] Potassium ranking: 66% [32] Phosphorous ranking: 85% [32] Transplant Qs: 0/4 error-free [25]; 5.45 ± 1.30/7 [36] Spanish translation linguistic accuracy: 4.89 ± 0.31/5 [35] Labs interpretation: median 2/5 (all labs), 4/5 (kidney labs) [19]	Urological malignancy Qs: 31.2 ± 12.6 [40]/11.7 (5.7–26.0) [41] Kidney stone Qs: 10.5 (10.0–11.3)/13.4 (12.1–15.9) [42] Kidney stone information: 14.0 ± 2.4 [45]; 5.4 (prevention), 6.7 (treatment) [44] Kidney donation Qs: 7.72 ± 1.85 [34]	Urological malignancy Qs: DISCERN 3 (2–4) [41] Kidney stone Qs: DISCERN 3 (3–4) [42] Kidney stone information: DISCERN 62 (prevention), 52 (treatment) [44] CKD Qs: GQS 1.93 ± 0.63/5 [37]	Spanish transplant Qs: 4.96 ± 0.19/5 [35]	Kidney cancer Qs: 3.9/5 empathy [39] Kidney stone Qs: outperformed urologists by mean empathy difference of 5.852–6.566 (*P* < .001) [27]; PEMAT understandability 69.2% and 63.3%, actionability 40% [42] Transplant Qs: 4.11 ± 1.96/5 recognizes feelings; 3.46 ± 2.05/5 understands situations [36] Urological malignancy Qs: PEMAT understandability 66.7%, actionability 40% [41]	N/A	Potassium ranking: 81% test–retest concordance [32] Phosphorous ranking: 90 % test–retest concordance [32]	Kidney cancer Qs: 4.1/5 tangibility and structural solidity, 3.4/5 reliability, 3.2/5 latest knowledge reflectivity, 3.7/5 assurance [39] Kidney stone Qs: outperformed urologists by mean completeness difference of 6.38–7.61 and mean practicality difference of 6.41–6.85 (*P* < .001) [27] CKD Qs: precision and recall F1 0.7–0.89 [37]
GPT-4	CRRT alarms: 92% [23] AKI/CRRT Qs: 97–98% [38] RCC Qs: 77.5% [26] Kidney stone Qs: 80%/93.3% [29] Kidney stone information: 1.5–3/5 [45] Oxalate ranking: 52% [33] Potassium ranking: 81% [32] Phosphorus ranking: 77% [32] Spanish translation linguistic accuracy: 4.94 ± 0.23/5 [35]	Kidney-stone information: 11.6 ± 2.3 [45] Kidney donation Qs: 4.30 ± 1.71 [34]	Kidney cancer Qs: GQS 68.1% [43]	Spanish transplant Qs: 4.96 ± 0.19/5 [35]	N/A	EAU urolithiasis: 4.80 ± 0.47/5 compliance [28] KDIGO CKD: alignment via RAG [24]	Kidney stone Qs: improved from 1.58 ± 0.515 to 2.83 ± 0.937/6 [29]	NDI diagnosis: specificity enhanced with CoT prompting [24] Kidney stone Qs: completeness 2.37 of 3/2.63 of 3 [29]
Perplexity	N/A	Urological malignancy Qs: 11.8 (7.4–38.1) [41] Kidney stone Qs: 10.8 (9.9–13.2)/12.6 (8.6–14.4) [42]	Urological malignancy Qs: DISCERN 5 (3–5) [41] Kidney stone Qs: DISCERN 4 (3–5)/5 (5–5) [42]	N/A	Urological malignancy Qs: PEMAT understandability 57.8%, actionability 30% [41] Kidney stone Qs: PEMAT understandability 75% and 73.3%, actionability 33.3% and 16.7% [42]	EAU urolithiasis: 4.68 ± 0.80/5 [28]	N/A	N/A
Bing	Oxalate ranking: 60% [33] Potassium ranking: 81% [32] Phosphorus ranking: 89% [32]	Urological malignancy Qs: 11 (4.9–36.6) [41] Kidney stone Qs: 12.1 (9.5–14.9)/10.7 (7.8–13.4) [42]	Urological malignancy Qs: DISCERN 5 (3–5) [41] Kidney stone Qs: DISCERN 4 (3–5)/5 (4–5) [42]	N/A	Urological malignancy Qs: PEMAT understandability 73.9%, actionability 40% [41] Kidney stone Qs: PEMAT understandability 77.8% and 70%, actionability 40% [42]	EAU urolithiasis: 4.21 ± 0.96/5 [28]	N/A	N/A
Bard AI	Oxalate ranking: 84% [33] Potassium ranking: 79% [32] Phosphorus ranking: 100% [32]	N/A	N/A	N/A	N/A	EAU urolithiasis: 3.56 ± 1.14/5 [28]	N/A	N/A
Copilot	Labs interpretation: 5/5 [19]	N/A	CKD Qs: GQS 2.02 ± 0.69/5 [37]	N/A	N/A	N/A	N/A	CKD Qs: Precision and recall F1 0.5–1 [37]
Gemini	Labs interpretation: median 3/5 (all labs), 4/5 (kidney labs) [19]	N/A	CKD Qs: GQS 3.46 ± 0.55/5 [37]	N/A	N/A	N/A	N/A	CKD Qs: precision and recall F1 0.8–1 [37]
Chat Sonic	N/A	Urological malignancy Qs: 12.2 (6.3–15.7) [41] Kidney stone Qs: 10.6 (9.3–12.4)/11.9 (10.1–14.7) [42]	Urological malignancy Qs: DISCERN 5 (3–5) [41] Kidney stone Qs: DISCERN 4 (3–5)/5 (3–5) [42]	N/A	Urological malignancy Qs: PEMAT understandability 63.3%, actionability 40% [41] Kidney stone Qs: PEMAT understandability 66.7% and 50.5%, actionability 20% and 0% [42]	N/A	N/A	N/A

GPT: generative pretrained transformer; RCC: renal cell carcinoma; Qs: questions; PEMAT: Patient Education Materials Assessment Tool; N/A: not applicable; EAU: European Association of Urology; NDI: nephrogenic diabetes insipidus.

**Table 6:** Comparison of advantages and limitations of LLM modalities.

Clinical application	Ref.	Potential advantages of LLMs	Limitations of LLMs
Workflow optimisation	[23]	High accuracy in interpreting CRRT alarms High consistency GPT-4 outperformed ChatGPT Potential to reduce ICU alarm fatigue GPT-4 can potentially solve newly encountered alarms	Human verification still required Findings are specific to CRRT; not yet applicable to other critical care devices Further training needed for broader clinical scenarios (including rare conditions) Potential for bias and data quality issues Cannot fully replicate clinicians' complex decision-making Lack of real-time integration with CRRT alarms limits timely intervention in ICU settings Empirical validation needed to bridge experimental results with practice
	[24]	CoT enables more specific diagnoses and mimics physician reasoning, especially for multistep or rare conditions RAG accesses external literature and guidelines to support evidence-based medicine Customizable profiles for nephrologists	CoT and RAG require complex prompt engineering and manual updates Only GPT-4 was evaluated Effectiveness is limited by the pretraining data Ethical and legal concerns: constant verification needed to detect bias or hallucinations Further enhancements needed for generalizability
	[25]	Accessibility concerns Relatively accurate basic concepts Consistency over sessions close apart	Fabrications in responses and references, with inconsistent evidence support Shallow domain-specific understanding Risk of misinformation, ethical and scientific integrity concern No verifiability Lack of clinical reasoning abilities Consistency across sessions may indicate limited contextual flexibility
	[26]	Improved accuracy through fine-tuning GPT-4 outperformed ChatGPT Potential as an auxiliary clinical tool Binary responses are efficient	Risk of providing inaccurate information, which may be detrimental in oncology Shallow reasoning and considering several treatment options in a binary response Both ChatGPT and GPT-4 showed instability and inaccuracies in their responses Data cut-off does not allow adherence to updating guidelines Further training and validation still required Limited generalizability to more complex medical tasks or other domains
	[27]	Relevant and accurate responses to patient inquiries, with highly rated empathy Instant access to medical sources Potential in reducing healthcare burden	Yet to replicate human reasoning, deficient understanding of non-verbal cues, sociocultural considerations, etc. Empathy is algorithmic and based on pattern replication rather than real-time understanding Limited responses due to training data gaps Lack of real-world clinical reasoning, further refinement is needed for more personalised care Risk of hallucinations Further validation is required prior to introduction into actual clinical setting Possibly confusing responses due to information overload and relevance issues There was no statistically significant agreement across raters in most parameters evaluated
	[28]	High accuracy of responses Can adhere to current guidelines Relatively consistent performance across several domains	May provide unreliable information. Currently, no validated tool exists to verify response accuracy May not always refer to official guidelines as a source Performance may fluctuate over time Limited generalizability into other domains
	[29]	High accuracy of both binary and descriptive responses High adaptability, able to improve responses over time Consistent performance across questions of different difficulty levels (according to Kruskal–Wallis tests) and types (binary and descriptive)	Quality of responses remains unsatisfactory, especially in more complex cases Limited generalizability to other domains Cannot learn or access new data post-training; limited by static knowledge base Should not replace clinical judgement
Laboratory data interpretation	[19]	No ethical concerns with simulated patient data Copilot outperformed other models Copilot provided detailed responses Statistical analysis suited for nephrology	GPT-4 not evaluated Variable response lengths Copilot relies on online sources, limiting its use for complex medical data Limited evaluation of 10 simulated patients Further training and validation needed Subjective rating of responses Lack of individualization Fluidity in accuracy over time
Renal dietary guidance	[32]	High accuracy in classifying potassium and phosphorus content High consistency Potential to reduce healthcare workload through automation GPT-4 shows advancements over ChatGPT Can assess various dietary items	Inconsistency in results remains, inconsistent phosphorus classification Clinical validation needed to avoid misinformation and patient harm Recommendation accuracy depends on input quality Lack of personalized dietary recommendations Ethical/legal concerns with clinical AI use
	[33]	Bard AI demonstrated higher accuracy, consistent across categories Potential aiding in personalized nutrition	Accuracy and consistency across categories are still suboptimal, may be unreliable ChatGPT tends to provide repetitive outputs ChatGPT and GPT-4 showed delayed response when given >300 prompts Performance may fluctuate over time Yet to be validated in real-world clinical setting
Patient education	[34]	Promotes equity, reducing healthcare disparities Utilizes online content patients can access Accuracy and fidelity confirmed Two independent sessions in new chats were conducted to assess reproducibility GPT-4 simplifies information well ChatGPT likewise improves accessibility for broader demographics	ChatGPT (free) less consistent than GPT-4 (paid) Limited to kidney donation FAQs Potential for regression in readability with updates Flesch–Kincaid formula may miss readability complexities
	[35]	Promotes health equity, addressing language barriers High cultural sensitivity and accuracy No significant differences between ChatGPT versions Uses FAQs from reputable sources High inter-rater reliability (Cohen's κ = 0.85)	Subjective scoring system Limited to Spanish translation; further evaluations needed for other languages (and other medical areas) Only two LLMs tested Occasional lower translation scores
	[36]	Reflects real-world concerns about kidney transplants from Reddit High perceived quality and empathy	Subjective scoring by non-professionals Only ChatGPT evaluated; version unspecified Perception varies by race and education Limited generalizability beyond kidney transplant
	[37]	High accuracy and precision for ChatGPT and Gemini Strong recall performance across modalities	Moderate quality for Gemini Performance varies between models and question types Inadequate compared with reference; potential misinformation Study limited to CKD questions Question sources not noted
	[38]	High accuracy from GPT-4 across different formats, including misspellings and incomplete sentences Consistent across CRRT and AKI topics Reliable for patient education High reliability (Cronbach's α = 0.94); each question was tested in a new chat session to prevent model adaptation Capable of providing accessible medical information to individuals with varying literacy levels	Free version of ChatGPT not evaluated Limited to nephrology topics Cannot replace doctor–patient interactions; further research needed for real-world application
	[39]	Generally acceptable and understandable responses in the context of kidney cancer Improves accessibility to basic medical information for the public	Cannot replicate the depth of clinical expertise held by practicing physicians Lower trust among specialists: expert urologists rated ChatGPT significantly lower on assurance compared with general urologists (*P* = .028) Outdated knowledge base: limited to pre-2021 data, lacking updates from recent guidelines Potential for unreliable or overly generic information, particularly on nuanced or complex topics
	[40]	Baseline readability was not inferior to established health information sources Improved readability with adjustment to audience Potential in enhancing health literacy	Inconsistent responses that are hard to regulate for clinical use Provided information may be outdated Can't substitute clinician guidance; lack of non-verbal and contextual elements critical for understanding
	[41]	High-quality information with no detected misinformation Responses urge users to seek professional care	Cancer treatment information quality showed lower scores High reading level of provided responses LLMs did not provide visual aids Responses are brief (median word count of 100), may limit comprehension
	[42]	High-quality information with no detected misinformation Most LLMs used frequent citations of reputable medical sources	High reading level of provided responses Unclear generalizability Limited by last major training update, may not comply with newly published guidelines Most LLMs did not provide visual aids
	[43]	High-quality responses to FAQs More reliable than social media Helpful for public awareness	Unsatisfactory responses for guideline-based questions Ethical and legal issues in incorporation into healthcare Information may be outdated: may not follow recent guideline updates Lack of personalized recommendations Lack of professional oversight over provided information; risk of spreading misleading information Many users use the free version of ChatGPT
	[44]	Generated responses are easier to understand compared with online search results Good quality of responses regarding kidney stone prevention and treatment Easy to use and highly accessible Holds potential to improve health literacy	No detailed discussion on management options, especially surgical ones Limited generalizability
	[45]	High accuracy Responses can refer to official resources Slight improvement of readability when specifically asked to respond at a 6th-grade level Potential as an adjunct for patient education	Even with prompt adjustments, readability still above recommended threshold No source transparency Reliance on online sources may cause inaccurate responses Omissions of details, especially for surgical management of kidney stones Lack of visual aids for better comprehension Can't substitute for experienced physicians
	[46]	Potential in increasing patient compliance with medical recommendations Provided satisfactory answers for most questions Strong repeatability of responses Can access variable sources	Performance may vary over time Continuous monitoring of response quality and relevance is required

GPT: generative pretrained transformer; ICU: intensive care unit.

### Quality assessment, risk of bias and reporting standards evaluation

To evaluate reporting quality, we applied the TRIPOD-LLM checklist. Although most studies adequately described background context and study objectives, substantial shortcomings were observed in multiple domains. Information on data handling and dataset characteristics was frequently incomplete, resulting in only partial fulfilment of the data-related items. Details of model implementation and prompting strategies were often omitted. Moreover, open-science practices, including code sharing and reproducibility measures, were seldom reported. Finally, few studies addressed fairness, bias or the practical deployment context of the LLMs. A summary of the TRIPOD-LLM assessment appears in Table 7.

**Table 7:** TRIPOD-LLM.

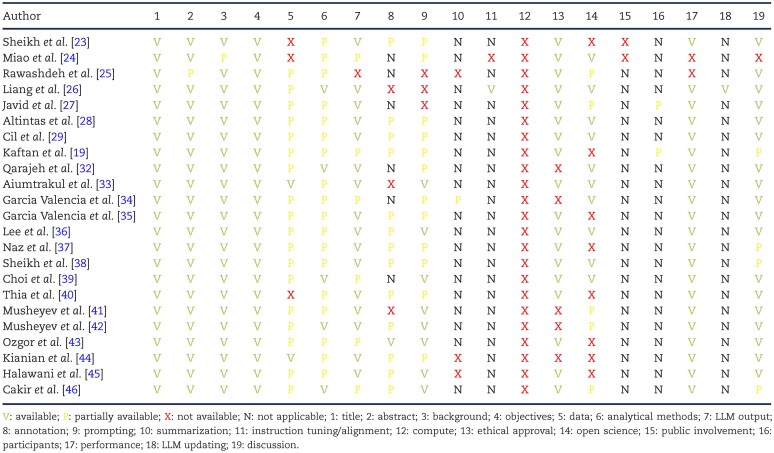

To assess the quality and risk of bias, we employed an adapted version of the QUADAS-2 tool across the 23 included studies. The detailed quality assessment is provided in Supplementary Material 3.

### Descriptive summary of the results according to the four clinical applications

#### Workflow optimization

Seven studies were classified in this domain, each addressing distinct aspects of nephrology practice [23–29].

Sheikh *et al.* [23] demonstrated the high accuracy of GPT-4 (90–94%) in managing continuous renal replacement therapy (CRRT) alarms, surpassing ChatGPT (84–86%); however, the difference was not statistically significant. Given that the CRRT questions were intended to reflect typical scenarios, the authors suggested that LLMs may help reduce alarm fatigue and potentially improve patient safety.

Miao *et al.* [24] described the application of GPT-4 to queries from practicing nephrologists, using a nephrogenic diabetes insipidus (NDI) diagnosis as an exemplar. They evaluated chain of thought (CoT) prompting, which guides the model to articulate step-by-step reasoning [30], and retrieval-augmented generation (RAG), which incorporates external evidence into the response [31]. These approaches increased diagnostic specificity and improved concordance with Kidney Disease: Improving Global Outcomes (KDIGO) guidelines.

#### Laboratory data interpretation

Kaftan *et al.* [19] investigated the performance of three LLMs in interpreting 10 simulated nephrology laboratory datasets. Copilot achieved the highest accuracy. Its advantage over ChatGPT was statistically significant (*P* = .002 for all results; .001 for kidney function tests), as was its advantage over Gemini (*P* = .008; .005 for kidney function tests). Importantly, GPT-4 was not assessed, and the analysis included only 10 cases. While Copilot performed well, it depends on input quality and derives its responses from online sources that may not be specifically curated for clinical use.

#### Renal dietary guidance

The renal diet, an essential component of kidney disease management, was examined in two studies [32, 33]. Qarajeh *et al.* [32] evaluated four LLMs for their ability to classify foods by potassium and phosphorous content. GPT-4 and Bing Chat showed the highest accuracy for potassium (81%), whereas Bard AI achieved perfect accuracy for phosphorous (100%). Aiumtrakul *et al.* [33] similarly assessed these models for oxalate classification. Bard AI again obtained the best overall accuracy (84%), followed by Bing Chat (60%), GPT-4 (52%) and ChatGPT (49%). The authors noted that accuracy decreased as task complexity increased, particularly when foods contained moderate or high oxalate levels.

#### Patient education

The ability of LLMs to answer patient inquiries and concerns was described in 13 studies [34–46]. Garcia Valencia *et al.* [34, 35] contributed two studies to this group, both highlighting the potential of LLMs to support inclusivity and equity in renal care. In the first study [34], they evaluated the capacity of ChatGPT and GPT-4 to simplify 27 frequently asked questions (FAQs) about kidney donation. Each model was tested twice in separate chat sessions. GPT-4 lowered the average reading grade level from 9.6 ± 1.9 (≈10th grade) to 4.30 ± 1.71 (≈4th grade), whereas ChatGPT reduced it to 7.72 ± 1.85 (≈8th grade). The goal was to simplify the text to below an 8th-grade reading level. GPT-4 achieved this in 96.3% of cases, while ChatGPT succeeded in 59.26% of cases, suggesting that the free ChatGPT model may provide more limited readability support than GPT-4.

In the second study, Garcia Valencia *et al.* [35] assessed the English-to-Spanish translation of 54 kidney transplant FAQs. Two Spanish-speaking nephrologists rated the translations and reported high linguistic accuracy (ChatGPT: 4.89 ± 0.31; GPT-4: 4.94 ± 0.23) and cultural sensitivity (4.96 ± 0.19 for both models). Unlike the simplification task, no significant differences were observed between the paid and free ChatGPT versions (linguistic accuracy: *P* = .26; cultural sensitivity: *P* = 1.00).

Five additional articles examined the readability of LLM-generated patient education material [40–42, 44, 45]. Kianian *et al.* [44] and Halawani *et al.* [45] focused on kidney stone education. Both studies found that prompt engineering—specifically instructing the model to write at a 6th-grade reading level—partially improved readability. Nonetheless, improvements were inconsistent, and in the work of Halawani *et al.* the text often remained above the recommended grade level.

Lee *et al.* [36] evaluated ChatGPT responses to 86 kidney transplant questions drawn from Reddit. Information quality and empathy were rated by 565 survey participants. Although clinicians were not explicitly excluded, the study sought lay perceptions of the responses. Among non-White respondents, higher education predicted higher perceived quality [mean = 6.03, standard error (SE) = 0.39], whereas among White respondents higher education predicted lower perceived quality (mean = 5.85, SE = 0.39).

Similarly, Naz *et al.* [37] analysed the accuracy and quality of information supplied by three LLMs. The models were asked to answer 40 FAQs reflecting parents’ concerns about chronic kidney disease (CKD). Two independent paediatric nephrologists rated the generated responses against KDIGO guidelines. ChatGPT and Gemini both exhibited comparatively high accuracy for diagnosis and CKD lifestyle questions. Among the evaluated models, Gemini achieved the highest average Global Quality Score (GQS; 3.46 ± 0.55).

## DISCUSSION

In this systematic review, we examined the use of various LLMs in nephrology and kidney disease. Our analysis centred on four domains of patient care and considered how these tools may support clinical workflows and patient engagement. Nevertheless, current LLM implementations are constrained by input-quality dependency and a lack of external validation across diverse clinical settings.

### Applications from the physicians’ perspective

LLMs such as GPT-4 have demonstrated a potential to streamline nephrology workflows, particularly in the intensive care setting. Yet their use in tasks such as CRRT alarm management still lacks robust external validation and prospective real-world evaluation [23].

Musheyev *et al.* [41, 42] found that LLM chatbots generally provided accurate information; however, accuracy decreased when they tackled complex, guideline-based questions, yielding lower DISCERN scores, particularly in treatment topics. Likewise, Ozgor *et al.* [43] and Cakir *et al.* [46] reported that ChatGPT performed better on FAQ-style queries than on detailed, guideline-derived questions. Several studies [39, 40, 42, 43] also noted that the static knowledge base of current LLMs may produce a lag in reflecting updated clinical guidance. Based on these findings, we propose integrating RAG techniques to better align LLM outputs with up-to-date clinical guidelines and enhance information reliability.

Conventional LLMs primarily rely on pretrained knowledge from large datasets, which may limit their capacity to deliver up-to-date, evidence-based medical recommendations. In contrast, RAG could expand LLM functionality by dynamically retrieving current information from structured databases, clinical guidelines and medical literature before responses are generated. This approach may allow the model to integrate current medical knowledge into its outputs, thereby reducing the risk of outdated content and hallucinations [47]. For example, Miao *et al.* [24, 31] reported that adding RAG improved alignment with KDIGO guidelines and may support diagnostic decision-making in nephrology. Recent work has also described the potential value of RAG for renal nutrition [48]. Nevertheless, additional validation is required to verify the reliability of retrieved data, and continuous updates are necessary to keep pace with evolving clinical standards. Ethical concerns surrounding cloud-based processing of patient data and information security remain significant barriers to widespread clinical adoption.

LLMs may contribute to disease prediction by helping forecast CKD progression and acute kidney injury (AKI), thereby potentially facilitating early interventions. Models like STRAFE and AKI-BERT leverage unstructured clinical data to identify high-risk patients and may enhance personalized patient management [49, 50].

Unlike the LLMs discussed in this review, which focus on language processing and conversational interaction, STRAFE is a transformer-based model designed for survival analysis and time-to-event prediction [49]. Its emphasis on disease progression modelling distinguishes it from conventional LLM approaches and underscores the broader applicability of transformer architectures in nephrology.

While these developments show promise, the ability of tools such as ChatGPT to simulate a physician's thought process remains limited. Although LLMs can mimic diagnostic reasoning by articulating step-by-step logic, these models still require further validation before being fully integrated into clinical practice [24, 30]. For instance, Kaftan *et al.* [19] showcased Copilot’s accuracy in interpreting 10 laboratory-value sets; however, its dependence on online resources and its limited ability to handle complex medical data indicate that further real-world validation is necessary. Despite their potential, the generalizability of current models remains restricted and additional evidence is needed before widespread clinical adoption.

A key methodological limitation across the included studies is the lack of external validation. Although many tasks showed promising LLM performance, the evaluations were conducted outside real-world clinical environments. This absence substantially limits the generalizability and clinical applicability. As LLMs advance toward patient care integration, future research should prioritize prospective validation to ensure safe and reliable implementation in healthcare settings.

### Applications from the patients’ perspective

The incorporation of LLMs into nephrology holds potential to bridge gaps in patient education and increase access to medical information. For instance, GPT-4 can simplify complex medical concepts, offer culturally sensitive translations and answer FAQs—even when queries contain misspellings or are incomplete [34, 35, 38]. Most of the reviewed studies analysed LLM responses to real-world online inquiries that patients might encounter when searching for nephrology information. These studies suggest that LLMs could enhance the readability and reach of health-related content across literacy levels and languages, thereby supporting efforts toward inclusivity and health equity [34, 35]. A notable performance gap persists between the paid and free ChatGPT versions: GPT-4 consistently achieved better readability scores in simplification tasks [34]. Although this difference concerns language simplification only—and does not necessarily reflect overall information quality and comprehension outcomes—it raises questions about equitable access to plain-language health content. Such concerns are particularly relevant for users limited to free tools.

LLMs may also oversimplify information, potentially omitting critical details required for comprehensive patient understanding [51]. Lee *et al.* [36] observed that patient perception of LLM responses can vary, suggesting that LLM output may not always be uniformly accessible or comprehensible to all patients. Moreover, because LLMs lack face-to-face interaction, they are limited in their empathy and personalization, which are fundamental to effective doctor–patient communication [38]. These limitations underscore ethical concerns regarding their integration into clinical settings.

### Limitations

Our systematic review focused on four areas of potential clinical application of LLMs in nephrology and explicitly excluded non-clinical use cases. Several of the included articles relied on relatively small datasets. The heterogeneity of clinical tasks and study methods among the selected articles prevented a meta-analysis. Because the field of LLMs is rapidly evolving, firm conclusions still require additional evidence.

Another limitation is the predominant focus of existing studies on technical metrics, such as accuracy or language performance, rather than on patient-centred outcomes. Further investigations should examine whether LLMs can actually improve clinical decision-making, patient care or health results. As AI applications expand, future work should prioritize real-world impacts on measurable health outcomes.

Additionally, the absence of a structured conceptual framework for comparing studies makes it challenging to derive overarching insights. Developing such a framework could potentially help to identify clinically meaningful outcome measures, align AI research with nephrology practice needs and guide the responsible adoption of LLMs in nephrology care.

In conclusion, although LLMs hold promise at multiple levels of nephrology practice, widespread implementation is premature. Further studies are needed to validate their accuracy, performance in rare and critical conditions and effectiveness in real-world settings.

## Supplementary Material

sfaf243_Supplemental_File

## Data Availability

All data related to this review is included in the published article and its supplementary materials. Any additional data are available upon reasonable request to the authors.
