# Supplementary material for: Clinical applications and limitations of large language models in nephrology: a systematic review
Source: Clin Kidney J. 2025 Sep 18;18(9):sfaf243. doi: 10.1093/ckj/sfaf243 (PMC12461145; doi:10.1093/ckj/sfaf243)
Supplement: sfaf243_Supplemental_File [file sfaf243_supplemental_file.docx]

**Supplementary Materials**

**Table of Contents:**

Supplementary Material 1- Overview of AI Modalities………………………………………………2

Supplementary Material 2- Literature Search…………..……………………………………...……….5

2.A) PubMed Search

2.B) Web of Science Search

2.C) Cochrane Library Search

2.D) Embase Search

Supplementary Material 3- Quality Assessment of Diagnostic Accuracy Studies (QUADAS-2)……...8

1. **Overview of AI Modalities**

*Artificial Intelligence (AI)* aims to train a computer to perform tasks usually requiring human cognition. AI is a general term referring to a broad range of models (1).

*Natural Language Processing (NLP)* is an important domain within AI, offering various functions related to human language. NLP allows for human language understanding, such as human-like interactions between the user and the chatbot, text generation and processing, and many other functions (2,3).

*Deep Learning (DL)* is an advanced type of AI. Within NLP it is used to facilitate its complex linguistic functions. The underlying DL architecture is inspired by the function of biological neurons. It is based on artificial neural networks arranged in multiple layers (hence “deep”). Data processing is handled by interconnected nodes representing neurons, where each "neuron" is similar to a single logistic regression unit (4).

When the user presents the chatbot with a textual input (termed prompt), the text then passes through several layers of interconnected nodes, each layer allowing the algorithm additional understanding of the text. An *attention mechanism* is used, detecting individual importance of words within a sentence (5). This ultimately allows the machine to understand the written text in a contextual manner, and therefore, more accurately.

*Transformers in AI* are a specific type of multi-layered neural network used in DL, characterized by their use of attention mechanisms (6). A major advancement in transformers occurred with the release of *Bidirectional Encoder Representations from Transformers (BERT)* (7,8). BERT improved the application of transformer architecture and achieved state-of-the-art results in various NLP tasks. The improvement offered by BERT was due to several factors, including its bidirectional text processing ability, its pre-training and fine-tuning processes, and its effective adaptability to new tasks.

*Large Language Models (LLMs)* represent a significant development in the field of transformers and the expansion of NLP’s capabilities. LLMs enable complex language generation skills, as seen in well-known chatbots such as openAI’s Chat Generative Pre-Trained Transformer (ChatGPT) (9) and Google’s Gemini (10). These platforms allow users to pose prompts, and receive written, coherent, and contextual answers generated by AI. When the prompt is more descriptive, the generated answer becomes more accurate.

Language generation by LLMs is based on predicting the most probable sequence of words, one by one. LLMs are trained on very large databases and then fine-tuned via reinforcement learning—a process of improving the tool’s performance through its own experience. The powerful text analysis capabilities offered by LLMs can be widely used across numerous professions, potentially alleviating the burden of intricate data processing and information retrieval, thus enabling a more effective workflow.

OpenAI offers both free and paid versions of ChatGPT, with the free version utilizing GPT-3.5 and the paid version powered by the more advanced GPT-4. In this paper, ChatGPT will refer to the GPT-3.5 version, while GPT-4 will denote the paid, more advanced model to maintain clarity between the two versions.

As AI continues to evolve, its applications in various medical fields are becoming increasingly prominent (11–19). This systematic review aims to explore how the diverse functions of LLMs can be leveraged to enhance clinical care within the field of nephrology.

**References:**

1. Klang E. Deep learning and medical imaging. *J Thorac Dis* 2018;10:1325–1328.

2. Sorin V, Barash Y, Konen E, Klang E. Deep-learning natural language processing for oncological applications. *Lancet Oncol* 2020;21:1553–1556.

3. Radford A, Wu J, Child R, Luan D, Amodei D, Sutskever I. Language Models are Unsupervised Multitask Learners [Internet]. 2019. [cited 2025 Jun 12]. Available from: <https://cdn.openai.com/better-language-models/language_models_are_unsupervised_multitask_learners.pdf>

4. Soffer S, Ben-Cohen A, Shimon O, Amitai MM, Greenspan H, Klang E. Convolutional Neural Networks for Radiologic Images: A Radiologist’s Guide. *Radiology* 2019;290:590–606.

5. Vaswani A, Shazeer N, Parmar N, et al. Attention is all you need. In: Guyon I, Luxburg UV, Bengio S, Wallach H, Fergus R, Vishwanathan S, Garnett R, eds. Advances in Neural Information Processing Systems 30 (NeurIPS 2017). Red Hook, NY: Curran Associates; 2017:5998–6008.

6. Dosovitskiy A, Beyer L, Kolesnikov A et al. An Image is Worth 16x16 Words: Transformers for Image Recognition at Scale. **arXiv:2010.11929** [Preprint]. 2020 [cited 2025 Jun 12]. Available from: <https://arxiv.org/abs/2010.11929>

7. Gorenstein L, Konen E, Green M, Klang E. Bidirectional Encoder Representations from Transformers in Radiology: A Systematic Review of Natural Language Processing Applications. *J Am Coll Radiol*2024;21:914–941.

8. Anil R, Dai AM, Firat O et al. PaLM 2 Technical Report. **arXiv:2305.10403 [Preprint]. 2023 [cited 2025 Jun 12].** Available from: <https://arxiv.org/abs/2305.10403>

9. Brown TB, Mann B, Ryder N et al. Language Models are Few-Shot Learners. In: Larochelle H, Ranzato M, Hadsell R, Balcan MF, Lin H, eds. Advances in Neural Information Processing Systems 33. Red Hook, NY: Curran Associates;2020:1877–1901.

10. Gemini Team, Georgiev P, Lei VI et al. Gemini 1.5: Unlocking multimodal understanding across millions of tokens of context. **arXiv:2403.05530 [Preprint].** 2024 [cited 2025 Jun 12]. Available from: <https://arxiv.org/abs/2403.05530>

11. Sorin V, Barash Y, Konen E, Klang E. Large language models for oncological applications. *J Cancer Res Clin Oncol* 2023;149:9505–9508.

12. Sorin V, Glicksberg BS, Artsi Y et al. Utilizing large language models in breast cancer management: systematic review. *J Cancer Res Clin Oncol* 2024;150:140.

13. Artsi Y, Sorin V, Konen E, Glicksberg BS, Nadkarni G, Klang E. Large language models for generating medical examinations: systematic review. *BMC Med Educ* 2024;24:354.

14. Omar M, Soffer S, Charney AW, Landi I, Nadkarni GN, Klang E. Applications of large language models in psychiatry: a systematic review. *Front Psychiatry* 2024;15:1422807.

15. Mudrik A, Nadkarni GN, Efros O, Glicksberg BS, Klang E, Soffer S. Exploring the role of Large Language Models in haematology: A focused review of applications, benefits and limitations. *Br J Haematol* 2024;205:1685–1698.

16. Omar M, Brin D, Glicksberg B, Klang E. Utilizing natural language processing and large language models in the diagnosis and prediction of infectious diseases: A systematic review. *Am J Infect Control* 2024;52:992–1001.

17. Klang E, Sourosh A, Nadkarni GN. Evaluating the role of ChatGPT in gastroenterology: a comprehensive systematic review of applications, benefits, and limitations. *Therap Advs Gastroenterol* 2023;16:17562848231218618.

18. Barash Y, Klang E, Konen E, Sorin V. ChatGPT-4 Assistance in Optimizing Emergency Department Radiology Referrals and Imaging Selection. *J Am Coll Radiol* 2023;20:998–1003.

19. Glicksberg BS, Timsina P, Patel D et al. Evaluating the accuracy of a state-of-the-art large language model for prediction of admissions from the emergency room. *J Am Med Inform Assoc* 2024;31:1921–1928.

1. **Literature Search**

2.A) Pubmed Search:

**((((((((((((((((((((((((((kidney*) OR (renal)) OR (nephrology)) OR (kidney disease*)) OR (renal disease*)) OR (renal disorder*)) OR (nephropath*)) OR (acute kidney injury)) OR (acute renal failure)) OR (AKI)) OR (acute renal insufficiency)) OR (chronic kidney disease)) OR (CKD)) OR (chronic renal failure)) OR (chronic renal insufficiency)) OR (hemodialysis)) OR (dialysis)) OR (renal replacement therapy)) OR (glomerulonephritis)) OR (glomerulopath*)) OR (glomerular disease*)) OR (glomerular disorder*)) OR (nephrotic syndrome)) OR (nephritic syndrome)) OR (urinalysis)) OR (renal function test*)) AND ((((((((((large language model*) OR (LLM)) OR (ChatGPT)) OR (OpenAI)) OR (Microsoft bing)) OR (Google bard)) OR (Google gemini))) OR (BERT)) OR (transformer*))**

**2.B) Web of Science Search:**

TS=((kidney* OR renal OR nephrology OR "kidney disease*" OR "renal disease*" OR "renal disorder*" OR nephropath* OR "acute kidney injury" OR "acute renal failure" OR AKI OR "acute renal insufficiency" OR "chronic kidney disease" OR CKD OR "chronic renal failure" OR "chronic renal insufficiency" OR hemodialysis OR dialysis OR "renal replacement therapy" OR glomerulonephritis OR glomerulopath* OR "glomerular disease*" OR "glomerular disorder*" OR "nephrotic syndrome" OR "nephritic syndrome" OR urinalysis OR "renal function test*") AND (("large language model*" OR LLM OR ChatGPT OR OpenAI OR "Microsoft bing" OR "Google bard" OR "Google gemini" OR BERT OR transformer*)))

2.C) Cochrane Library Search:

**kidney OR renal OR nephrology OR "kidney disease" OR "renal disease" OR "renal disorder" OR nephropath OR "acute kidney injury" OR "acute renal failure" OR AKI OR "acute renal insufficiency" OR "chronic kidney disease" OR CKD OR "chronic renal failure" OR "chronic renal insufficiency" OR hemodialysis OR dialysis OR "renal replacement therapy" OR glomerulonephritis OR glomerulopath OR "glomerular disease" OR "glomerular disorder" OR "nephrotic syndrome" OR "nephritic syndrome" OR urinalysis OR "renal function test" in Title Abstract Keyword AND "large language model" OR LLM OR ChatGPT OR OpenAI OR "Microsoft Bing" OR "Google Bard" OR "Google gemini" OR BERT OR transformer in Title Abstract Keyword - with Cochrane Library publication date Between Jan 2020 and Jul 2024 (Word variations have been searched)**

- As this search was conducted retrospectively, and given system limitations, it was not possible to match the exact search date within July 2024 for searches 2.A, 2.B and 2.D.

2.D) Embase Search:

('nephrology'/exp OR 'nephrology' OR 'kidney'/exp OR 'kidney' OR 'renal disease'/exp OR 'renal disease' OR 'kidney disease'/exp OR 'kidney disease' OR renal:ti,ab OR kidney*:ti,ab OR nephrology:ti,ab OR 'kidney disease*':ti,ab OR 'renal disease*':ti,ab OR 'renal disorder*':ti,ab OR nephropath*:ti,ab OR 'acute kidney injury':ti,ab OR 'acute renal failure':ti,ab OR aki:ti,ab OR 'acute renal insufficiency':ti,ab OR 'chronic kidney disease':ti,ab OR ckd:ti,ab OR 'chronic renal failure':ti,ab OR 'chronic renal insufficiency':ti,ab OR hemodialysis:ti,ab OR dialysis:ti,ab OR 'renal replacement therapy':ti,ab OR glomerulonephritis:ti,ab OR glomerulopath*:ti,ab OR 'glomerular disease*':ti,ab OR 'glomerular disorder*':ti,ab OR 'nephrotic syndrome':ti,ab OR 'nephritic syndrome':ti,ab OR urinalysis:ti,ab OR 'renal function test*':ti,ab) AND ('large language model*':ti,ab OR llm:ti,ab OR chatgpt:ti,ab OR openai:ti,ab OR 'microsoft bing':ti,ab OR 'google bard':ti,ab OR 'google gemini':ti,ab OR bert:ti,ab OR transformer*:ti,ab) AND [01-01-2020]/sd NOT [22-07-2024]/sd

1. **Quality Assessment of Diagnostic Accuracy Studies (QUADAS-2)**

To assess the quality and risk of bias, we employed an adapted version of the QUADAS-2 tool across the 23 included studies. Overall, most studies exhibited a moderate-to-high risk of bias for the index test, predominantly owing to the absence of external validation and ambiguity regarding the independence of index-test interpretation from the reference standard. Four studies were rated high risk of bias in at least one domain. The data-management domain likewise showed a moderate-to-high risk of bias in most studies, mainly because of incomplete or poorly specified data-handling procedures and the absence of explicit safeguards for data integrity.

| Author / risk of bias | Patient selection | Index test | Reference standard | Flow and timing | Data management |
| --- | --- | --- | --- | --- | --- |
| Sheikh et al. | N/A | X* | V** | N/A | V |
| Miao et al. | N/A | M* | M | N/A | V |
| Rawashdeh et al. | N/A | X* | V** | N/A | M |
| Liang et al. | N/A | M* | V | N/A | V |
| Javid et al. | V | V | M | N/A | M |
| Altintas et al. | N/A | M* | V | N/A | V |
| Cil et al. | N/A | M* | V | N/A | M |
| Kaftan et.al | X | M | V | V | X |
| Qarajeh et al. | N/A | M | V | N/A | M |
| [Aiumtrakul](https://pubmed.ncbi.nlm.nih.gov/?term=Aiumtrakul+N&cauthor_id=38248809) et al. | N/A | M* | V | N/A | V |
| Garcia Valencia et al. | N/A | M* | V | N/A | M |
| Garcia Valencia et al. | N/A | M* | V | N/A | M |
| Lee et al. | M*** | M* | M | N/A | M |
| Naz et al. | N/A | M* | V | N/A | M |
| Sheikh et al. | N/A | M* | M | N/A | M |
| Choi et al. | N/A | M* | M | N/A | V |
| Thia et al. | N/A | M* | V | N/A | X |
| Musheyev et al. | N/A | V | V | N/A | M |
| Musheyev et al. | N/A | V | M | N/A | M |
| Ozgor et al. | N/A | M* | V | N/A | V |
| Kianian et al. | N/A | M* | V | N/A | M |
| Halawani et al. | N/A | M* | V | N/A | V |
| Cakir et al. | N/A | M* | V | N/A | V |

* Of note, the setting is experimental, **Not universally accepted, ***Voluntary online survey.

Index: V= low risk of bias, M=moderate risk of bias, X= high risk of bias, N/A= not applicable
